# Changes in Envelope Structure and Cell–Cell Communication during Akinete Differentiation and Germination in Filamentous Cyanobacterium *Trichormus variabilis* ATCC 29413

**DOI:** 10.3390/life12030429

**Published:** 2022-03-16

**Authors:** Ritu Garg, Manja Luckner, Jürgen Berger, Katharina Hipp, Gerhard Wanner, Karl Forchhammer, Iris Maldener

**Affiliations:** 1 Institute of Microbiology and Infection Medicine, Organismic Interactions, University of Tübingen, 72076 Tübingen, Germany; ritu.garg@uni-tuebingen.de (R.G.); karl.forchhammer@uni-tuebingen.de (K.F.); 2 Department of Biology I, Ludwig-Maximilians-University, 82152 Munich, Germany; manja.luckner@t-online.de (M.L.); wanner@lrz.uni-muenchen.de (G.W.); 3 Max-Planck Institute for Developmental Biology, Electron Microscopy, 72076 Tübingen, Germany; juergen.berger@tuebingen.mpg.de (J.B.); katharina.hipp@tuebingen.mpg.de (K.H.)

**Keywords:** cyanobacteria, *Trichormus variabilis*, akinetes, FIB/SEM tomography, cell-cell communication, FRAP

## Abstract

Planktonic freshwater filamentous cyanobacterium *Trichormus variabilis* ATCC 29413 (previously known as *Anabaena variabilis*) can differentiate heterocysts and akinetes to survive under different stress conditions. Whilst heterocysts enable diazotrophic growth, akinetes are spore-like resting cells that make the survival of the species possible under adverse growth conditions. Under suitable environmental conditions, they germinate to produce new vegetative filaments. Several morphological and physiological changes occur during akinete formation and germination. Here, using scanning electron microscopy (SEM), we found that the mature akinetes had a wrinkled envelope, and the surface of the envelope smoothened as the cell size increased during germination. Thereupon, the akinete envelope ruptured to release the short emerging filament. Focused ion beam–scanning electron microscopy (FIB/SEM) tomography of immature akinetes revealed the presence of cytoplasmic granules, presumably consisting of cyanophycin or glycogen. In addition, the akinete envelope architecture of different layers, the exopolysaccharide and glycolipid layers, could be visualized. We found that this multilayered envelope helped to withstand osmotic stress and to maintain the structural integrity. Furthermore, by fluorescence recovery after photobleaching (FRAP) measurements, using the fluorescent tracer calcein, we found that intercellular communication decreased during akinete formation as compared with the vegetative cells. In contrast, freshly germinating filaments restored cell communication.

## 1. Introduction

The filaments of the multicellular cyanobacterium *Trichormus variabilis* ATCC 29413 consist of hundreds of vegetative cells that have the potential to differentiate into specialized cells, heterocysts and akinetes. The semi-regularly spaced heterocysts enable the fixation of atmospheric nitrogen (N_2_), if no other combined nitrogen sources are available, and provide organic nitrogen to the vegetative cells [1,2]. Akinetes are spore-like nonmotile single cells that develop from the vegetative cells in response to diverse environmental factors, including light intensity, temperature and nutrient deficiency [3,4,5,6]. They are resistant to different types of biotic and abiotic stresses, compared with vegetative cells [4,7].

Akinete differentiation from vegetative cells involves the development of overall-different structures, which includes cell enlargement, granulation and, similar to heterocysts, development of a thickened multilayered envelope surrounding the cells [8,9,10,11]. Akinetes contain abundant reserves of nitrogen, stored in cyanophycin bodies, and carbon, in glycogen granules [4,12,13,14]. However, it was recently shown that the production of cyanophycin granules is not crucial for akinete development and germination in *T. variabilis* [6]. Although the akinete stage is considered dormant, minimum metabolic activities were observed in mature akinetes [9,15]. The content of DNA and RNA in akinetes of species *Nostoc* PCC 7524 were similar to those of vegetative cells [16]. The transient resistant akinete structure allows cyanobacteria to survive harsh environmental situations, and they can undergo germination after favorable conditions return [1,4]. Light, moderate temperature and nutrient conditions suitable for growth appear to be the major stimuli for akinete germination [4,5,7,8,9,17,18,19,20].

The akinete envelope is composed of several distinct layers consisting of exopolysaccharides and glycolipids [9,10,21,22]. Heterocyst glycolipids (HGs), present in many heterocyst-forming cyanobacteria, were also identified in the akinetes of *Cyanospira rippkae* [23,24,25,26]. In *T. variabilis*, a lipid layer was detected in the akinete envelope, which was composed of the same glycolipids (HG_26_-diol) that formed the heterocyst envelope [22]. Furthermore, the *hglB* gene is responsible for the synthesis of this lipid for both heterocyst and akinete envelopes [27].

Akinetes are highly resistant to various stress factors, such as darkness, osmotic stress, desiccation, freezing and a wide range of temperatures [4,16,17,28,29,30,31,32,33]. In consequence, akinetes can survive buried in sediments for several decades [34]. The akinetes of *Nostoc* sp. HK-01 have tolerance to dry heat due to the accumulation of compatible solute glucosylglycerol, betaine and glycine [35,36]. Therefore, akinete formation is considered a key strategy for survival under extreme conditions and is responsible for perennation in several orders of cyanophycean.

The multicellular lifestyle including cell differentiation and division of labor along the filaments in heterocyst-forming cyanobacteria requires mechanisms of cell–cell communication. Cells must perceive signals and communicate with each other through direct cell–cell connections, the septal junctions, which allow the intercellular exchange of metabolites and regulatory compounds to be performed [37,38,39]. The septal junctions are gated proteinaceous complexes that resemble metazoan gap junctions [40]. In the septal-cell walls, an array of nanopores (circular perforations) drilled by AmiC-type amidases were discovered [41,42,43,44]. These nanopores are the framework for the formation of septal-junction complexes between adjacent cells [43]. The gating of septal junctions depends on environmental conditions, as shown by measuring the cell–cell-communication rates under stress conditions using fluorescence recovery after photobleaching (FRAP) measurements [39,40].

Since environmental signals trigger several vegetative cells in the trichomes of *T. variabilis* to differentiate into akinetes, it can be assumed that regulatory molecules are exchanged among cells during akinete induction. During differentiation, structural, cellular and physiological changes occur, leading to a complete separation of the cell. After sensing unfavorable conditions, the cells of the filament start to build an extra envelope, which, finally, surrounds each individual cell. At the same time, developing akinetes reduce their metabolic activities and require less communication with each other. Exactly the opposite happens when akinetes start to germinate; on their way to multicellular filaments, cell–cell communication restarts, enabling the formation of semi-patterned heterocysts. However, nothing is known about the dynamics of cell–cell communication during akinete differentiation and germination. Here, we addressed the question of whether the exchange of metabolites and signaling molecules correlates to their physiological conditions during these two processes. We used the fluorescent marker calcein to follow the molecule exchange during akinete differentiation and germination. Furthermore, we demonstrated, by scanning electron microscopy and FIB/SEM tomography, several changes in the cell structure occurring during akinete formation and germination to provide a basis for understanding the survival strategy of akinetes in the model organism, *T. variabilis* ATCC 29413.

## 2. Materials and Methods

### 2.1. Strains and Growth Conditions

*Trichormus variabilis* ATCC 29413 strain FD [45,46] was cultivated photoautotrophically under continuous illumination (17–22 μmol photons m^−2^ s^−1^) in Erlenmeyer flasks at 28 °C with shaking at 120 rpm in BG11 medium containing NaNO_3_ [47] or on medium solidified with 1.5% (*w*/*v*) Difco Agar.

### 2.2. Akinete Induction and Germination

Akinete differentiation was induced in the late phase of an exponentially growing culture by transferring the filaments to low-light conditions (2–3 μmol photons m^−2^ s^−1^) by covering the culture flasks with paper towels [9]. Akinete-induced cultures were maintained at 28 °C with gentle shaking at 50 rpm.

The germination of mature akinetes that had been kept in low light from two to three months (hereafter called 2–3-month-old akinetes) was induced by washing and transferring the culture to either BG11 medium (containing NaNO_3_) or BG11_0_ medium lacking combined nitrogen and optimal light conditions [22].

Akinete differentiation and germination was observed using a Leica DM 2500 light microscope with an 100x/1.3 oil objective, connected to a Leica DFC420C camera (Leica Microsystems GmbH, Wetzlar, Germany).

### 2.3. Osmotic-Stress Resistance

From a liquid culture of vegetative filaments and a suspension of 2–3-month-old akinetes, 1 mL was centrifuged, and the supernatants were removed. After the addition of 200 µL of 40% sucrose, the cells were incubated at room temperature (RT) for 20 min, then immediately visualized under a bright-field microscope. For SEM, cells were fixed after sucrose treatment with 4% formaldehyde and 2.5% glutaraldehyde in 1x PBS for 2 h at RT followed by overnight incubation at 4 °C. Cells were washed in PBS, post-fixed with 1% OsO_4_ on ice for 1 h and transferred onto poly-L-lysine-coated coverslips. After gradual dehydration in ethanol and critical-point drying (CPD300; Leica), the samples were sputter-coated with a 3 nm platinum layer (CCU-010; Safematic) and examined with a Hitachi Regulus SU 8230 field emission scanning electron microscope (Hitachi High Technologies, Tokyo, Japan) at an accelerating voltage of 5 kV.

### 2.4. Fluorescent Recovery after Photobleaching (FRAP) Assay

For FRAP, the samples were collected during akinete differentiation and germination at different time points. The loading of *T. variabilis* vegetative cells and akinetes with calcein and the FRAP measurements were performed as previously described [37,48]. Briefly, cells were washed three times and resuspended in 500 µL of fresh BG11 medium; this was followed by the addition of 10 µL of calcein acetoxymethylester (1 mg/mL in DMSO) and incubation in the dark for 2.5 h at 28 °C with gentle agitation. The samples were washed again three times with BG11 medium, then further incubated for 1.5 h with gentle shaking in the dark. The cell suspensions were spotted onto BG11 agar and covered with a cover slip. All FRAP measurements were performed at RT with a Zeiss LSM 800 confocal microscope using a 63x/1.4 oil-immersion objective and ZEN 2.3 (blue edition) software as described previously [44]. The 488 nm line of a 10 mW laser at 0.2% intensity was used as the excitation source. Chlorophyll *a* auto-fluorescence (emission detection: 650–700 nm) and calcein fluorescence (emission detection: 400–530 nm) were imaged simultaneously using a 191 µm confocal pinhole (corresponding to 4.49 airy units) resulting in a point spread in the Z-direction of about 3 mm.

For imaging, the following settings were used: laser intensity, 0.2%; frame size, 36.2 × 36.2 µm; pixel size, 0.07 µm; pixel dwell time, 1.52 µs; averaging, 1x line average. After capturing five initial prebleach images, the laser intensity was increased by a factor of at least 10 for bleaching the fluorescence of a region of interest by a ‘fast-bleach’ option. A sequence of images at 1 s intervals for 30–60 s was taken to record the recovery of the fluorescence signal in the bleached cell. The images were processed using the ‘Time series analyzer V3′ ImageJ plugin for measuring the fluorescence intensity of a FRAP sequence, and data were processed with GraphPad PRISM v6.01 for Windows (GraphPad Software, La Jolla, San Diego, CA, USA). The fluorescence recovery rate constant *R* of a bleached cell was calculated as described previously using the formula C_B_ = C_0_ + C_R_ (1 − e^−2*Rt*^), where C_B_ is the fluorescence of the bleached cell, C_0_ is the fluorescence immediately after the bleach and tending towards (C_0_ + C_R_) after fluorescence recovery, C_R_ is the fluorescence during recovery, *t* is time, and *R* is the recovery rate constant due to the molecular exchange of the tracer with neighboring cells [42,48].

### 2.5. EM Preparation and FIB/SEM Imaging

A culture that had been incubated for akinete differentiation for two months contained mature akinetes and was induced to germinate. Samples of 500 µL were collected before germination and at different time points during germination. The cells were fixed with 2.5% glutaraldehyde in cacodylate buffer (2 mM MgCl_2_, 50 mM cacodylate; pH 7.0) for 30 min at RT followed by overnight incubation at 4 °C. The cells were postfixed with 1% (*v*/*v*) OsO_4_ and 1% (*w*/*v*) K_4_[Fe(CN)_6_] in cacodylate buffer for 30 min, washed 3 times in ddH_2_O, incubated with 1% (*w*/*v*) thiocarbohydrazide in ddH_2_O for 30 min, washed 3 times with ddH_2_O, then postfixed with 1% OsO_4_ in ddH_2_O for 30 min. The samples were rinsed 3 times with ddH_2_O before being dehydrated in a graded series of acetone containing a step of 1% uranyl acetate in 20% acetone for 30 min, infiltrated and ultrathin-embedded on glass slides, then imaged by focused ion beam–scanning electron microscopy (FIB/SEM) tomography as described previously [49].

### 2.6. Three-Dimensional Reconstruction and Visualization

The datasets were aligned using Amira™ version 2019.2 (Thermo Fisher Scientific, Waltham, MA, USA) with the module *align slices*. The FIB/SEM-image stacks were segmented and 3D-reconstructed or processed with a direct volume rendering algorithm (VOLREN) for immediate visualization.

## 3. Results

### 3.1. Morphological Changes Associated with Akinete Formation

The transition from dividing vegetative cells to dormant akinetes is associated with morphological changes and the accumulation of storage compounds [9]. Under standard laboratory growth conditions, akinete differentiation was induced in stationary-phase cultures of *T. variabilis* by transferring the filaments to low-light conditions and maintaining them under low light for 2 months. The cultures of *T. variabilis* began to differentiate akinetes after 3–7 days of incubation, and 95 % of the cells had differentiated into akinetes after 60 days. Akinete differentiation was characterized by the visual change in color of the cultures from blue–green to yellow–brown and by the fragmentation of the filaments into oval-shaped mature akinetes (Figure S1A) [9].

**Figure 1:**
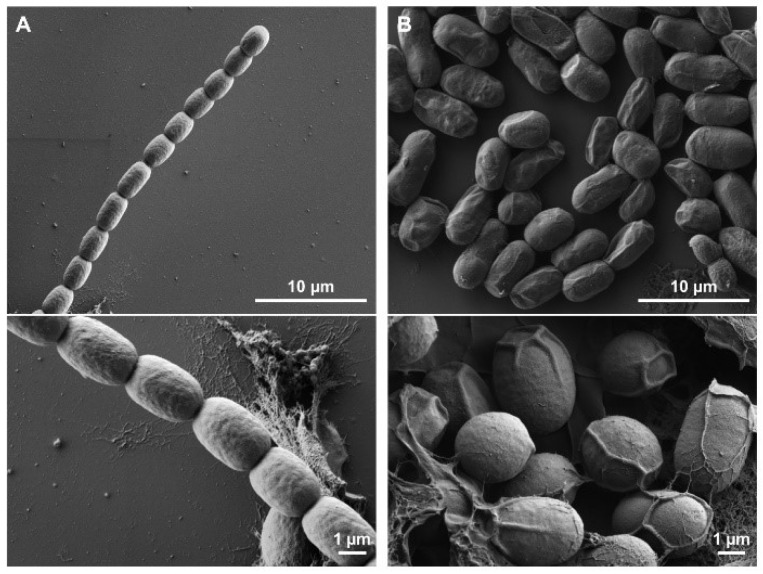
Scanning electron micrographs of *Trichormus variabilis*. (**A**) Young vegetative filament. (**B**) Two-month-old mature akinetes induced under low-light conditions.

**Figure 2:**
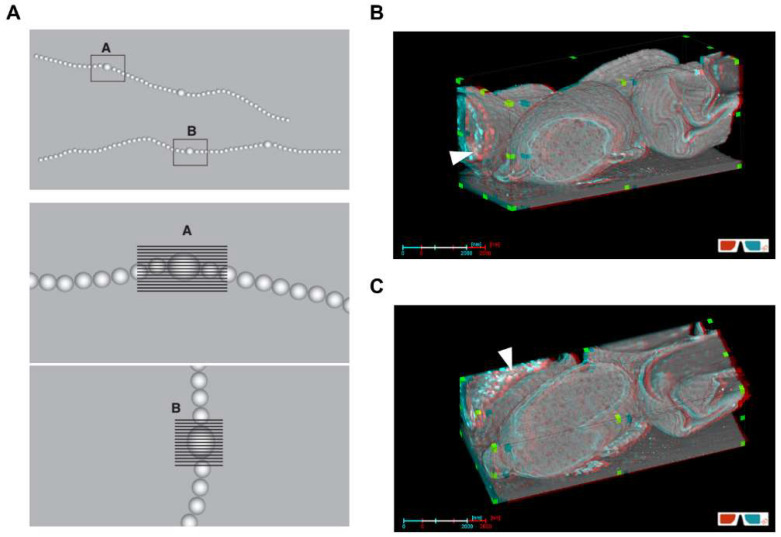
Three-dimensional visualization of akinetes from FIB/SEM tomogram of *T. variabilis*. (**A**) Illustration of FIB-milling process with *T. variabilis* filament after flat embedding in SEM. Box denotes the targeted cells for FIB-miling; shaded parts indicate horizontal or vertical cross-sectioning of cells. (**B**,**C**) SEM images of two-month-old akinete after longitudinal (**B**), then transversal (**C**) FIB milling. White arrowheads indicate the cytoplasmic granules in immature akinete. Use of 3D glasses is recommended.

To elucidate the morphological changes during akinete differentiation, scanning electron microscopy was performed. As shown in Figure 1, the surface of the vegetative cells was smooth and waveless (Figure 1A), while mature akinetes had wrinkles or folds on their surface (Figure 1B). To understand the structure of akinetes in more depth, focused ion beam (FIB)/SEM tomography [50] was used, where akinetes were cut in sections first vertically, then horizontally (Figure 2A). The cross-sections created by FIB milling were further analyzed by FIB/SEM tomography. The akinetes showed, without exception, typical envelopes and cellular structures (Figure 2B,C). A thick envelope coat formed around the cell, consisting of several layers that were folded in multiple places on the outer surface (Figure 2C). This multilayered envelope composed of several distinct layers was also shown by previous studies using transmission electron microscopy [9]. Notably, the FIB/SEM images depicted that the folds or wrinkles were only present in the envelope. Many immature akinetes also showed intracellular granules, which disappeared once the akinete reached its mature stage (Figure 2B,C and Figure S1B); this is consistent with a previous study that showed that younger akinetes (18 days) accumulated glycogen and cyanophycin granules, which disappeared in older akinetes (30 days) [9].

### 3.2. Tolerance of Akinetes against Sucrose Treatment

**Figure 3:**
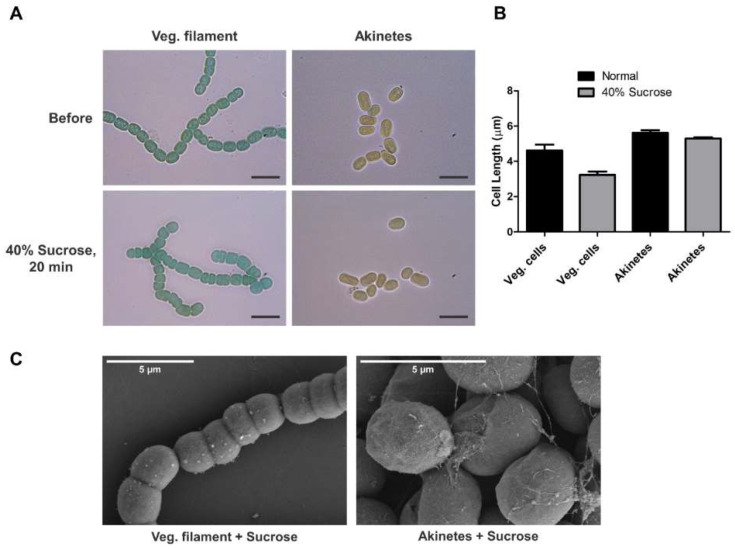
Akinetes are resistant to osmotic stress. (**A**) Bright-field images of vegetative filaments and akinetes treated with 40% sucrose for 20 min. Scale bar, 10 µm. (**B**) Cell-length measurement using ImageJ before and after sucrose treatment of vegetative cells and akinetes. (**C**) SEM images of vegetative filaments and akinetes after treatment with 40% sucrose.

Akinetes are the key for survival under harsh environmental conditions, where they must face various drastic changes. To understand the protective capability of akinetes, the limit of tolerance of akinetes against osmotic stress was investigated. For this, vegetative filaments and akinetes were treated with 40% sucrose and monitored under a bright-field microscope for 20 min. The results showed a drastic decrease in the filament length due to shrinkage of the vegetative cells, while no effects on akinete dimensions were observed (Figure 3A). The length of approximately 90–100 cells in the filaments and akinetes was measured using ImageJ, showing that the akinetes were able to maintain their structure and size of individual cells during sucrose treatment; however, the vegetative cells in the filaments showed a 30 % shrinkage longitudinally, from a mean length of 4.62 ± 0.16 µm to 3.23 ± 0.09 µm (Figure 3B), while no effects on cell width were observed. We also observed normal germination of akinetes after sucrose treatment (not shown).

Next, we analyzed the morphological changes in the cells after sucrose treatment by SEM (Figure 3C). The length of the vegetative cells treated with sucrose was reduced, resulting in shorter filaments than those of the untreated ones (Figure 1A and Figure 3C). However, the surface of the vegetative cells was not affected by the sucrose treatment. Akinetes showed no obvious changes in their dimensions nor morphologies (Figure 1B and Figure 3C), consistently with the bright-field micrographs. Our results indicate that the akinete envelope kept its structure; this may help in the maintenance of structural integrity, which is confirmed by the normal germination efficiency of the akinetes that we observed after sucrose treatment.

### 3.3. Analysis of Akinete Germination Process by SEM

**Figure 4:**
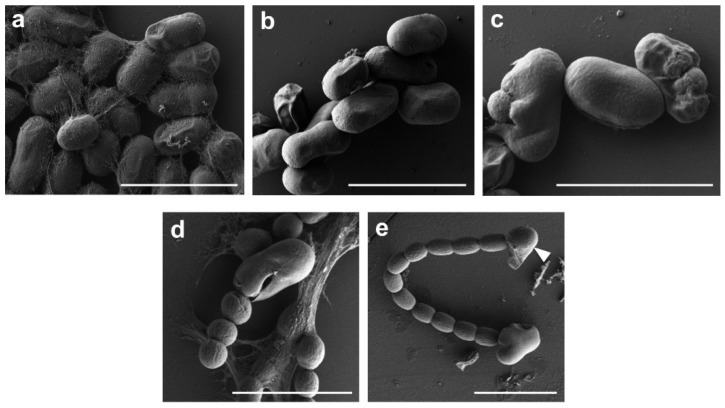
SEM analysis of akinete germination and cellular growth of *T. variabilis* at different time intervals. (**a**) Mature akinetes before germination; (**b**) akinete germination on day 1; (**c**) germinated filament inside the envelope on day 2; (**d**) cellular growth and trichome development on day 3, with attached envelope; (**e**) growing filament with terminal heterocyst differentiation in BG11_0_ medium on day 3. White arrowhead points to the terminally differentiated heterocyst. Scale bar, 10 μm.

The remarkable surface structure of the mature akinetes (Figure 1) with wrinkles and thick raised folds prompted us to investigate these surface structures during germination. The germination of akinetes was induced by transferring mature akinetes to fresh medium and optimum light conditions. The beginning of germination could be recognized by the increased volume of the akinetes, and the first cell division was visible between 17 h and 24 h. After 24 h, the increase in akinete size led to the disappearance of the wrinkles from the envelope (Figure 4b and Figure S2). Then, the akinete coat ruptured, and the small developing filament penetrated the akinete envelope mostly at one pole (Figure 4c). The release of two–four-celled germlings by lysis of the akinete coat could be seen within 48 h (Figure 4d). Akinetes did not have a hole or any kind of aperture in their envelope surface (Figure S2), as it was observed for the coat of many bacterial spores [51]. After 72 h, the normal growing filaments were also seen to be still attached to the envelope of the akinetes (Figure 4d,e). Since media lacking a nitrogen source were used, we could also follow terminal-heterocyst formation during germination and heterocyst development, which was visible after approximately 48 h (Figure 4e).

### 3.4. Intercellular Communication during Differentiation and Germination of Akinetes

In previous studies, FRAP analyses showed that filamentous cyanobacteria exchange fluorescent tracers between the cytoplasm of the cells along filaments [37]. In addition, the transfer of tracers was observed between heterocysts and vegetative cells [37,42,52]. This indicates cell–cell communication along filaments and between heterocysts and vegetative cells.

**Figure 5:**
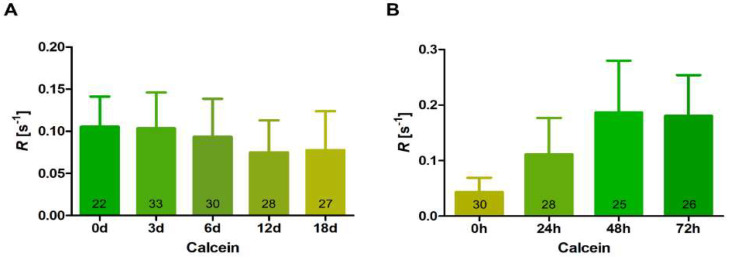
Fluorescence recovery after photobleaching (FRAP) analysis of the intercellular exchange of calcein during akinete differentiation and germination. (**A**) Cell–cell communication during akinete differentiation in low light over a span of 18 days. Numbers in bars indicate the number of analyzed cells (n) from different filaments subjected to FRAP analysis. Data are mean ± SD from the results obtained from three independent cultures. (**B**) Regaining of cell–cell communication during akinete germination up to 72 h in fresh medium and optimum light conditions. Numbers within the bars indicate number of analyzed cells (n) from different filaments subjected to FRAP analysis. Data are mean ± SD from three independent cultures.

We performed FRAP experiments to investigate cell–cell communication during akinete formation and germination. Using the diffusible fluorophore calcein, we followed the ability of molecule transfer in filaments during akinete differentiation. After sensing low-light conditions, the program of akinete differentiation started in *T. variabilis*. At the beginning, the immature akinetes remained attached to each other; then, they completely detached after maturation and stopped direct cell–cell communication. The time course of ceased molecule exchange between maturing akinetes was followed using FRAP measurements performed at different time points up to 18 days of akinete differentiation. Our results show a gradual decrease in calcein transfer, indicating slower intercellular intracellular communication during akinete differentiation (Figure 5A). Compared with the beginning of the experiment, 18-day pre-akinetes had reduced communication by approximately 30%. This indicates that cells preparing for dormancy are metabolically less active and do not need to communicate.

In one month old akinete cultures, we observed few filaments that mostly contained immature akinetes which had not yet detached from each other. When these akinetes were transferred to fresh medium and optimal light conditions, nearly all of them started to germinate and divide along with the germination of mature akinetes. So, we performed FRAP measurements during the germination of akinetes especially in these filaments and investigated the transfer of calcein in the dividing filaments. We observed that, after 24 h of germination, the immature akinete-containing filaments resumed greening, started to divide fast and showed a faster transfer of calcein, indicating the regaining of cell–cell communication (Figure 5B). After 48 h, an even faster calcein transfer rate was observed because dividing cells became metabolically active; this continued till 72 h, when almost all the filaments reached the normal vegetative growing state (Figure 5B). Furthermore, we observed that the freshly dividing young filaments after germination (48 h/72 h, Figure 5B) had twice as much communication as compared with the vegetative cells in the stationary culture that had been used at the onset of the experiment (Figure 5A). These results indicate that molecule exchange is regulated during these two processes and depends on the physiological conditions.

## 4. Discussion

Bacterial cells can adapt to changing external conditions by various mechanisms, including morphological and physiological changes, to maintain their cellular structure. A key feature that contributes to the success of *T. variabilis*’ survival under extreme conditions is their ability to form highly resistant akinetes. When environmental conditions become favorable, the dormancy of akinetes is broken and germination occurs, giving rise to the filaments of vegetative cells.

With the availability of various techniques, we were able to have a closer look at the akinete differentiation and germination processes. We used scanning electron microscopy (SEM) to visualize the surface, shape and size of vegetative filaments and mature akinetes. The SEM images showed wrinkles on mature akinetes, suggesting akinete-envelope folding (Figure 1). We assume that these wrinkles provide the structural flexibility of these cells. When turgor pressure increased during germination, the wrinkles smoothed out, providing space for the emerging filament inside the envelope before rupture.

By using FIB/SEM tomography, we could obtain high-resolution-image stacks of the entire akinete cells in 3D, which allowed us to detect morphological changes at different time points during differentiation from vegetative cells and maturation. Intracellular electron-dense granules were still present in immature akinetes, and the typical akinete envelope was not developed yet. The envelope of mature akinetes consists of several layers [9,22]. The accumulation of reserve granules observed in immature akinetes was mainly of glycogen and cyanophycin granules (Figure 2B,C) [9]. The direct role of cyanophycin granules is still unknown in *T. variabilis*, but it was found that they are not required for akinete formation and germination [6].

In accordance with our study, akinetes were able to resist osmotic stress and maintain their structure during sucrose treatment, whereas young vegetative cells turned out to be more susceptible (Figure 3A). Akinetes are highly resistant to many environmental stresses, owing in part to the presence of a multilayered envelope structure. Recently, we found that the glycolipid layer of the akinete envelope is crucial for protecting the akinetes from freezing, desiccation and lytic attacks [27]. It was suggested that the exopolysaccharide layer present in akinetes plays a role in membrane stabilization during desiccation [53]. For maintaining rigidity of the akinete envelope and to provide stress tolerance, the presence of hapanoids was also found to be necessary [54]. Our results demonstrate the importance of the akinete envelope in providing structural stability under osmotic stress. This adaptation is crucial for surviving the harsh environmental conditions prevailing in nature. Another possibility to consider is a reduced water content of the cytoplasm of akinetes compared to vegetative cells, which would be similar to bacterial endospores [55]. By this, the akinetes were less prone to shrinkage during osmotic stress.

The vegetative cells are surrounded by outer and inner membranes, which are separated by the periplasmic space and a peptidoglycan (PG) layer. The behavior of vegetative filaments during sucrose treatment was interesting, as we observed the shrinkage of cells only longitudinally, which is indicative of the architecture of peptidoglycans. Apparently, in the transversal direction, the wall was much more rigid, whereas it was elastic in the longitudinal direction (Figure 3A).

SEM was used to visualize structural changes during the germination, emergence and outgrowth of the short filament. In *T. variabilis*, we observed that an increase in light intensity rapidly triggered the germination of akinetes [22], which was similarly reported in previous studies on the germination of akinetes in *Anabaena cylindrica* and *Anabaena variabilis* Kutzing [17,21]. We observed that the first cell division occurred inside the akinetes upon transfer from dark or low light to medium light (ca. 20 µmol photons m^−2^ s^−1^) (Figure 4b). During germination, as the cell size increased, we observed the disappearing of the envelope folds; this suggests that akinete envelope can adapt to different sizes and provide the structural integrity after the initial cell divisions at the beginning of germination. After reaching a certain size, it finally ruptured to release the short filament (Figure 4b). After 48 h, terminally differentiated heterocysts were observed, indicating that, from the very beginning of cell division, the presence/absence of a source of combined nitrogen could be sensed by the small germinating filament (Figure 4e).

In many bacteria, spore-coat-degrading enzymes such as CwlJ and SleB are known to play redundant roles in the degradation of the spore peptidoglycan cortex during germination [56,57,58]. However, so far, no such enzymes were discovered for akinete-envelope degradation during germination. So, it would be of future interest to identify the envelope-degrading enzymes in cyanobacteria.

In our study, we also measured cell–cell communication during akinete differentiation and germination. Decreased communication during differentiation can be expected, as cells go into a dormant stage where they do not need to exchange molecules (Figure 5A). In contrast, with resuscitation, cells need to return to an active metabolism to allow cell division and growth to be performed. In the emerging filament of *T. variabilis*, rapid cell divisions occurred, and cells concomitantly started cell–cell communication (Figure 5B). This is particularly essential when heterocysts are formed, and the nutrient status of the cells has to be communicated to assure the typical heterocyst pattern in the filaments. Additionally, the freshly germinating filaments communicated even faster than standard vegetative cells (Figure 5A,B). Faster communication was also observed in hormogonia, short motile filaments, which have functions in dispersal and symbiotic association in several filamentous cyanobacteria [59]. The higher communication rate in young filaments and hormogonia could be important to sense the favorable light and nutritional conditions and to provide the motility necessary for hormogonia to establish symbiotic associations with plants [60]. The development of cell–cell-communication machinery during akinete germination, including nanopore formation in the freshly formed septal peptidoglycan layer and the building of septal-junction complexes during the transition from the unicellular to the multicellular organism, should be a focus in future research on akinete germination.

## Supplementary Materials

The following supporting information can be downloaded at: https://www.mdpi.com/article/10.3390/life12030429/s1, Figure S1: Two-month-old akinetes of *T. variabilis*. (A) Light micrographs showing akinetes induced under low-light conditions. Images of bright field (left) and red auto-fluorescence (right). Bar, 25 µm. (B) FIB/SEM images of akinetes after FIB milling and 3D visualization (volume rendering). Intracellular granules are present in immature akinetes (right) and absent in mature akinetes (left), Figure S2: Germinating akinete showed increase in cellular size and random rupture of envelope during germination. White arrowheads indicate breaks and distortion of the akinete envelope.
