# Supplementary material for: Changes in Envelope Structure and Cell–Cell Communication during Akinete Differentiation and Germination in Filamentous Cyanobacterium Trichormus variabilis ATCC 29413
Source: Life (Basel). 2022 Mar 16;12(3):429. doi: 10.3390/life12030429 (PMC8953462; doi:10.3390/life12030429)
Supplement: Supplementary file 1 [file life-12-00429-s001.zip › life-1592880-supplementary.pdf]

life

MDPI

# Supplementary Material of Changes in Envelope Structure and Cell–Cell Communication during Akinete Differentiation and Germination in Filamentous Cyanobacterium *Trichormus variabilis* ATCC 29413

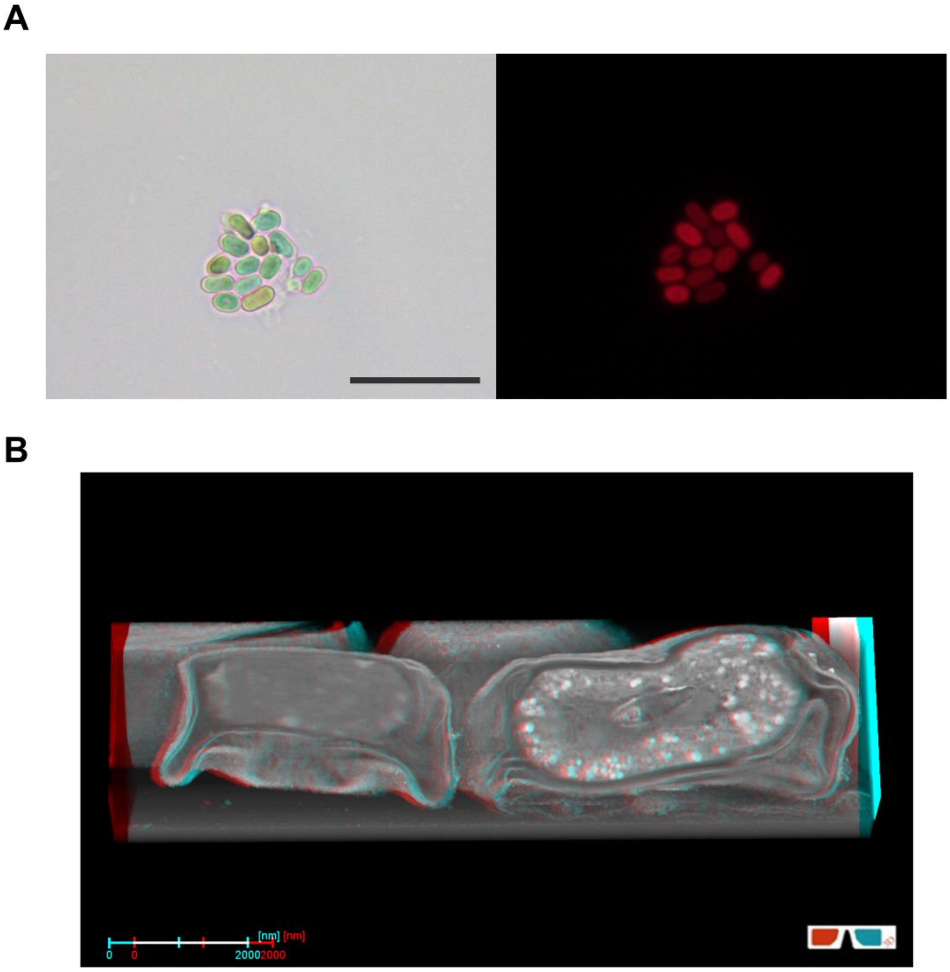


**Figure S1.** Two months old akinetes of *A. variabilis*. (**A**) Light micrographs showing akinetes induced under low light condition. Images of bright field (left), red auto-fluorescence (right). Bar, 25 µm. (**B**) FIB/SEM images of akinetes after the FIB milling and 3D visualization (volume rendering). Intracellular granules are present in immature akinete (right) and absent in mature akinete (left).

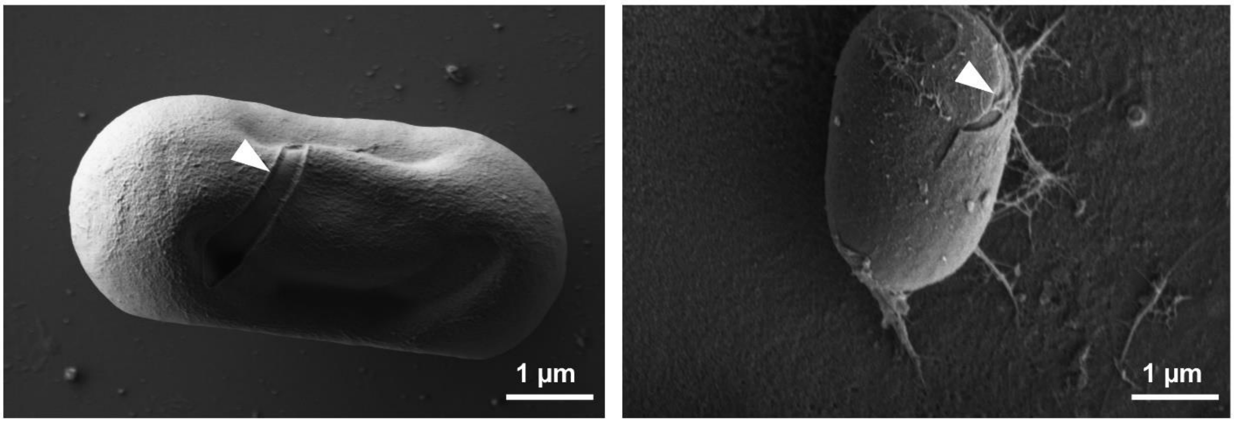


**Figure S2.** Germinating akinete showed increase in cellular size and rupture of envelope randomly during germination. White arrowheads indicate breaks and distortion of the akinete envelope.
